# Employment and work safety among 12 to 14 year olds: listening to parents

**DOI:** 10.1186/1471-2458-14-1021

**Published:** 2014-10-01

**Authors:** Amelia M Usher, Curtis Breslin, Ellen MacEachen, Mieke Koehoorn, Marie Laberge, Luc Laberge, Élise Ledoux, Imelda Wong

**Affiliations:** Institute for Work and Health, 481 University Avenue, Suite 800, Toronto, ON Canada; School of Public Health and Health Systems, University of Waterloo, Waterloo, ON Canada; School of Population and Public Health, University of British Columbia, Vancouver, BC Canada; School of Rehabilitation, Université de Montréal, Montreal, QC Canada; Department of Health Sciences, Université de Québec à Chicoutimi, Saguenay, QC Canada; L’Institut de recherche Robert-Sauvé en santé et en sécurité du travail, Montréal, QC Canada

**Keywords:** Canada, Parents, Odd jobs, Employment, Young adolescents, Occupational health, Job safety, Work hazard, Focus group

## Abstract

**Background:**

Survey research indicates that a surprising number of 12 to 14 year olds in North America engage in some form of paid work, and work-related injuries for this age group are reported at rates similar to older teens. Parents exhibit significant involvement in many aspects of their teens’ work and may influence perceptions of work safety, yet few studies have explored this phenomenon from a qualitative perspective with parents of working 12 to 14 year olds.

**Methods:**

This paper focuses on parental perceptions and understandings of work safety based on focus groups conducted with urban Canadian parents of young teens who work for pay. Parents discussed the types of job held by their 12 to 14 year olds, the perceived costs and benefits to working at this age, and their understanding of risk and supervision on the job. A grounded theory approach was used to thematically analyze the focus group transcripts.

**Results:**

Parents in this study held favourable attitudes towards their 12 to 14 year olds’ working. Parents linked pro-social moral values and skills such as responsibility, work ethic, time management, and financial literacy with their young teen’s employment experience. Risks and drawbacks were generally downplayed or discounted. Perceptions of workplace safety were mitigated by themes of trust, familiarity, sense of being in control and having discretion over their 12 to 14 year olds’ work situation. Further, parental supervision and monitoring fell along a continuum, from full parental responsibility for monitoring to complete trust and delegation of supervision to the workplace.

**Conclusions:**

The findings suggest that positive parental attitudes towards working overshadow occupational health and safety concerns. Parents may discount potential hazards based on the presence of certain mitigating factors.

## Background

In North America, a surprising number of 12 to 14 year olds work for pay outside their homes [1–4]. A school-based survey in Ontario found that just over half of 12 to 14 year olds in 2003 reported working for pay at some point during the school year [4]. British Columbia had somewhat lower rates, with 41.5% of 12 to 14 year olds working for pay during the school year [4]. A recent survey in Québec found that 47.9% of 12 to 14 year olds reported having worked for pay during the school year, although this included doing small chores at home for allowance as paid work, a type of work that other surveys tend to exclude [5]. Boys in Ontario and British Columbia tended to hold jobs in more formal work settings, which were defined as paid work in food service settings, retail stores, offices, or construction, while girls were more likely to work odd jobs such as babysitting [4]. In the United States (US), similar rates of paid work among young adolescents are reported. Surveys of middle school students in Texas indicate that close to 56% of sixth through eighth graders report working for pay [6]. Results of a survey in Massachusetts indicated that 1 in 4 middle school students reported working for pay in the last year, however this survey excluded babysitting and yard work [2].

Not only do many young adolescents work, they also perform a wide diversity of job tasks. A recent survey in Alberta, Canada of households with a 9 to 14 year old working for pay indicated that, although the majority were employed in babysitting and newspaper/flyer delivery, these youth also reported a range of other jobs such as janitorial, restaurant, and agricultural work [7]. While the work that young adolescents perform is largely classified as unskilled, Entwisle and colleagues [1] noted anecdotally that even 13 year olds in their urban US sample were asked to do some rather complex tasks such as carpentry, roofing, and plumbing.

More troubling is the increased potential for workplace injuries as younger teens begin working for pay. In a Québec sample, 11.3% of working 12 to 14 year olds reported some kind of work-related injury in the past 24 months [5], and studies in Ontario and British Columbia reported that 6.0% and 3.5% of 12 to 14 year olds respectively sought medical attention for a work injury [4]. It is notable that these work injury rates for 12 to 14 year olds are comparable to injury rates observed among 15 to 19 year old workers [8, 9]. In the last 10 years, the high young worker (i.e., 15 to 24 years of age) injury rates have been declining, and in Ontario and a few other jurisdictions have converged with adult work injury rates [10]. Nevertheless, some subgroups of youth (e.g., those who drop out of high school) remain at elevated risk [11]. In terms of what types of jobs might be most hazardous to young workers, a study of emergency department records for several Canadian hospitals showed that 82% of 10 to 13 year olds needing treatment for a work-related injury held a service or clerical job, with another 12% performing some kind of manual labour such as farming and construction [12]. Common work-related injuries sustained by youth under the age of 17 were upper extremity injuries, such as wounds to the hands and fingers, and animal bites [12]. This same study reported that two out of five work-related injuries presented at an emergency department during the summer, suggesting seasonal variation in work injuries as young adolescents may be more likely to be employed during the summer months.

Work is one of the primary ways that youth assert autonomy and demonstrate competence outside school and home [13]. Among older adolescents, some studies suggest that engaging in paid employment while still in school can have positive developmental implications for self-esteem, autonomy, skills acquisition, and psychosocial development [14–16]. Research has also identified disadvantages of paid work among high school students such as increased likelihood of alcohol consumption, cigarette smoking, and lower academic grades [17–19]. However, the impact of work among high school students appears to be influenced by contextual factors such as the families’ socioeconomic position, academic interest of the student, and the quality of work [20, 15]. Consequently, it is important to examine further the costs and benefits of work for 12 to 14 year olds, and the contextual factors that determine these costs and benefits.

Research with older teenagers suggests that many parents are quite involved in aspects of their teen’s work in ways that can influence the youth’s job selection and safety at work. For example, in a survey of parents of 14 to 18 year old workers in the US [21], almost 90% reported having helped their teen identify job opportunities and 46% had discussed a safety issue at their work with their teen. Many US parents believe that *parents* should determine the work their teen can do (69%) [22]. At the same time, they are also supportive of government regulations limiting the number of hours teenagers can work and laws prohibiting them from performing dangerous tasks. Despite this degree of parental involvement, there is evidence that parents lack specific knowledge of labour restrictions for young workers [23]. An important knowledge gap in the Canadian context is parents’ views of their 12 to 14 year old working and the parents’ understandings of their role regarding work safety for their daughter or son.

With regard to the legislative context, the International Labour Organization’s minimum age conventions state that member countries (which include Canada and the US) should prohibit paid work for youth under 14 years of age, although light work at age 13 that does not hinder school participation may also be permitted [24]. Legislation in Canada regarding minimum age restrictions for employment varies from province to province. For example, Ontario does not have a universal minimum age designation; rather age restrictions are industry-specific [25]. Fourteen year olds are allowed to work in industrial establishments such as offices, stores, arenas, and restaurant serving areas. The minimum age to work in a factory (which includes manufacturing, restaurant kitchens, automotive garages, warehouses, and food preparation) is 15 years old. Working in construction or logging operations is permitted starting at 16 years of age, while underground mines and window cleaning is permitted only for individuals aged 18 years and older. By comparison, the US Federal Fair Labor Standards Act has specific regulations prohibiting youth 17 years old or younger from operating certain equipment and machines identified as hazardous. In some States, these federal regulations are supplemented with additional restrictions on the hours youth can work and what work tasks they are allowed to perform [18].

Less attention has been given to the informal job sectors in which young adolescents are most commonly employed, such as babysitting and yard work [6, 3]. These informal jobs are often not specifically addressed in occupational health and safety regulations, leaving the possibility open that some 12 to 14 year olds could be considered self-employed. A common test for whether someone is considered an employee or self-employed includes criteria related to degree of control over work tasks and ownership of equipment used to carry out the work [26]. Whether or not a youth is considered self-employed would have important implications for who is responsible for the occupational health and safety of a 12 to 14 year old worker.

### Current study

There is a need to characterize the nature of work and occupational safety among 12 to 14 year olds given employment is relatively common in this age group and may lead to work injuries at about the same rate as older teens. As parents play a key role in work-related decisions at this age, it is particularly useful to understand their perspective on these issues. Based on the gaps identified in the literature, this study examines parent perceptions and understandings related to their 12 to 14 year olds’ work. More particularly, the study describes parent perceptions of cost and benefits, work safety, and supervision associated with their young teen’s work.

## Methods

### Study design

In keeping with the exploratory nature of the study, a qualitative research design was selected. Four focus groups were conducted in spring of 2013 with parents (*n* = 34) living in a large urban area in Ontario, Canada. Focus groups were adopted as the preferred method of eliciting perceptions and understandings in this study, as they provide the opportunity to capture a wide range of opinions and experiences while encouraging interaction between participants [27, 28].

### Recruitment and sample

Recruitment was undertaken by a market research company with established focus group facilities and a large consumer database of Canadian households. This firm has been used in a previous study on perceptions of workplace injury among young workers [29]. A recruitment screener was used to determine eligibility, which was limited to English-speaking parents with a child between the ages of 12 and 14 who worked for pay in the last 12 months. Participants were excluded if they had participated in a focus group within the past six months, worked for a media or public relations firm, or worked for an organization involved in workplace health and safety. Participants signed an informed consent form and a confidentiality agreement prior to the study and received an $80 incentive after completion of the focus groups.

A purposive sampling strategy was used such that participants would reflect a range of socio-economic and ethnic backgrounds (Table 1), as well as diversity of jobs held by their 12 to 14 year old children. Parents (*n* = 34) ranged in age from 28 to 59 years (*mean* = 41.9 years, *sd* = 7.9 years). Characteristics including age, gender, and job type of the 12 to 14 years olds (*n* = 36) discussed by participants are displayed in Table 2. The most commonly held jobs were newspaper/flyer delivery, babysitting, and yard work, followed by food sales and tutoring. The majority of parents indicated that their 12 to 14 year old worked during the school year (*n* = 30). Some parents indicated during the focus group discussions that their 12 to 14 year old had held more than one type of job in the past year, however this information was not directly asked of all parents during the recruitment process.

**Table 1:** **Characteristics of parent sample (n = 34)**

Characteristic	***n (***%***)***
Gender	
Male	13 (38.2)
Female	21 (61.8)
Ethnicity	
Caucasian	18 (52.9)
Black	9 (26.5)
Asian	3 (8.8)
South Asian	2 (5.9)
Arabic	2 (5.9)
Education Level	
High School	2 (5.9)
Some College	2 (5.9)
College	18 (52.9)
University	12 (35.3)
Household Income	
< $45,000	6 (17.6)
$45,000 – $60,000	8 (23.5)
$60,001 – $95,000	12 (35.3)
>$95,000	8 (23.5)

**Table 2:** **Parents’ report of the age, gender, and types of jobs held by their 12 to 14 year olds (n = 36)**

	Male (***n*** = 26)	Female (***n*** = 10)	Total (***n*** = 36)
Age			
12 years	7	4	11
13 years	9	5	14
14 years	10	1	11
Type of job			
Newspaper/Flyer delivery	8	2	10
Babysitting	4	4	8
Yard work	6	1	7
Food sales	1	1	2
Tutoring	2	–	2
Fast food outlet	1	–	1
Administration	1	–	1
Marina attendant	1	–	1
Convenience store	1	–	1
Dance instructor	–	1	1
Lifeguard	–	1	1
Camp counselor	1	–	1
Social Services	1	–	1
Racetrack	1	–	1
Hair salon	–	1	1
Painting	1	–	1
Dog walking	1	–	1
Cleaning	1	–	1

*Note*. Totals may exceed 36 as some 12 to 14 year olds were reported as having more than one type of job.

### Data collection

A total of four focus groups consisting of eight to ten people per group were conducted. These took place in the evenings over two days and lasted approximately 90 minutes each. The discussion guide contained a series of open-ended questions designed to elicit attitudes towards the benefits and drawbacks of their child working (e.g. "what are the good things that have come out of your son or daughter working? What are the not so good things?"), as well as parental perceptions of work safety and supervision (e.g. "what is your understanding of the supervision that your son or daughter receives while at work?"). Field notes were taken by the research associate and principal investigator. These notes were used to record seating arrangements, group dynamics, emerging issues for analysis, and salient contextual factors.

Focus groups were video recorded and transcribed in real-time by a transcriptionist. Transcripts were verified for accuracy against the video recordings by the research associate. Transcripts were cleaned of identifying information and participants’ names were changed to preserve confidentiality. Data was stored on a secure network where access was restricted to the research team. Ethical approval for this study was obtained from the University of Toronto’s Office of Research Ethics.

### Analysis

A modified grounded theory approach was used to analyze the focus group transcripts [30]. This type of analysis has four key steps: immersion in the data, coding, creation of categories, and explanation and interpretation of categories [31]. Immersion involves observing the groups and re-reading the transcripts. Coding refers to the sorting and tagging of units of meaning that can be added and redefined. Creation of categories requires examining ways to link codes in a systematic and coherent way. Grounded theory analysis techniques involve constant comparison and negative case analysis, which means continually assessing new text from the transcripts against understandings of what the analyst has learned from previous transcripts. Negative cases are those that do not ‘fit’ with trends in the findings. Negative cases were identified and further explored in subsequent data gathering and analysis in order to understand departures from the trend. These procedures of qualitative analysis ensure that the interpretations are supported by the data and help provide sufficient context for readers to make informed judgments as to their applicability to other groups and circumstances [32].

After the transcripts were checked for accuracy, they were subject to an initial round of coding using a set of thematic codes developed by the research team based on field notes and initial topics of interest. Themes capturing parents’ attitudes and perceptions towards their 12 to 14 year olds’ work were developed in an iterative fashion through a process of descriptive coding, reflecting back to the text, consultations with the research team, and combining codes into analytical themes. Quality and rigour of analysis was established through the following strategies: a) involving the research advisory team in the preparation of the discussion guide, b) taking detailed field notes during the focus groups, c) verifying accuracy of the transcriptions against original video recordings, d) undertaking multiple reviews of the coded transcripts by principal investigator and research associate in order to provide feedback on preliminary codes, and e) soliciting feedback from the research advisory team on the final set of themes generated through data analysis. Coding was conducted using Nvivo 10 software [33], a program that aids researchers in qualitative analysis.

## Results

Key themes emerged regarding how parents understood the hazards their 12 to 14 year olds encountered on the job, and how the perceived benefits of working served to overshadow job risks. To place these key themes in context, the many benefits and relatively few drawbacks to working identified by parents, as well as some common safety concerns, are described below.

### Benefits and drawbacks to working

Parents identified numerous benefits resulting from their 12 to 14 year old working for pay. Overall attitudes were positive, regardless of parent gender or type of job held by their 12 to 14 year old, and benefits were seen to outweigh any reservations held with respect to their young teens working. Parents expressed a strong desire for their children to learn general life skills such as responsibility, financial literacy, and time management and saw youth employment as a means to achieving these goals. Belief that working at this age would become a gateway to further employment opportunities down the road was also prominently discussed. These benefits seemed to reflect pro-social values that parents associated with engaging in paid work and which they actively wanted to foster in their young teens, as described by this mother:

*Just cause it teaches them so much. The value of working hard for your money, and making your own money, and being responsible, and having to go out there and do it when you don’t necessarily want to and you want to just sit around and play video games because he’s had a hard day at school.* (Mother of 12 year old son, newspaper/flyer delivery).

Certain drawbacks were also acknowledged such as neglecting school work and extra-curricular activities, fatigue, and stress. Concerns over wasteful spending, disrespectful behaviour toward parents (e.g. being mouthy or cocky), and not pursuing higher education were identified as well. Although parents were able to identify these drawbacks when probed, they did not feature prominently in the focus group discussions. The perceived benefits to working outnumbered and outweighed any identified downsides.

### Identified safety risks

By far the most common concern for parents was the risks of kidnapping and assault, as exemplified by these two parents:

*When your child is going door-to-door, any of those doors can be a bad door.* (Father of 12 year old son, social services).

*Just to add that, what if my daughter is babysitting and somebody comes to the door and she answers and god forbid something should happen? That is the safety issue I am talking about*. (Mother of 13 year old daughter, babysitting).

This view was particularly prominent among parents whose young teens worked in newspaper delivery and babysitting, which comprised a significant proportion of our sample. Interestingly, parents believed kidnapping and assault to be risks not only while their young teens were at work, but also during transit to and from work, regardless of the type of job. This was consistent across focus groups even though parents were specifically asked about safety at work, as opposed to safety in general. This suggests that parents see an overlap of the boundaries between safety concerns while at work and those in the broader environment for their young teens. It is noted that, when prompted, parents were able to identify some more common injury risks such as cuts, burns, or injuries sustained while operating equipment. However, these discussions were far outweighed by concerns about the risks to their young teens at the hands of strangers. Although parents were not directly asked whether their child sustained an injury, all groups were asked whether they had concerns about their child’s work safety, which provided an opportunity to mention any prevous injuries. There were three injuries reported across the four focus groups: a bite, a burn, and a laceration.

Although concerns about kidnapping and assault were discussed at length when probed, parents generally felt that their 12-14 year olds were safe at work. A number of themes emerged illustrating that concerns about occupational health and safety risks were overshadowed, and likely discounted, by parents. These themes centered primarily on familiarity, trust, and an overarching sense of being in control of their 12 to 14 year olds’ work situation.

### Familiarity and trust

Parents frequently expressed an implicit trust in their 12 to 14 year olds’ workplace or employer. Trust was often established through pre-existing personal relationships with neighbours or family members, who frequently served as employers, although trust was fostered in other ways as well. Familiarity with the job also stemmed from prior experience with similar jobs held by their older children or parents having held the same job in their own youth.

*In my case I knew the people involved that he was going to be working with, so the trust factor in terms of getting paid or not being asked to do more than is reasonable was ok. Just knowing the people involved was a lot of research in itself taken care of*. (Father of 12 year old son, dog walking and yard work)

*I used to do the paper route when I was younger too. And I go along with them and my mom used to come along with me. So I knew what it was because I had a feel for it.* (Mother of 12 year old daughter, newspaper delivery)

For parents whose 12 to 14 year old did not work in an environment that was already known, additional steps were taken so as to become familiar and establish trust. Examples of this included speaking to employers about onsite safety procedures and workload, or personally meeting the individuals who hired their children for babysitting and other odd jobs. This was particularly evident for informal work settings, where some parents stated that they would personally vet each babysitting job or dog walking client taken by their 12 to 14 year old.

*I talked to the manager before he started work and made sure of that. I wanted to know he was doing the training, how was the training happening. They showed me the manuals, the walk-throughs, I was assured that the first week he did go through training*. (Father of 14 year old son, fast food outlet)

*I think for babysitting, sometimes people just say "can your daughter babysit?" Ok, but who are you? I want to know everything […]. I think that the parents need to be more open. If I want to drop her off and come upstairs, I should be able to come upstairs with her and scope out the place.* (Mother of 12 year old daughter, babysitter)

### Discretionary nature of work

Parents expressed conditional support for their 12 to 14 year old working in terms of being able to balance their other responsibilities. As noted by one father of a 13 year old son, "*it was a precondition to maintain good grades*". Others echoed these sentiments, suggesting that work was perceived as a privilege that could be withdrawn or renegotiated by the parent at any time.

*Most of the work for my son is in the summer, but during the year he babysits or does yard work. So he can’t take those on unless his homework is done. There is a lot of negotiation.* (Mother of 14 year old son, camp counsellor)

The decision to engage in paid work was parent-initiated in some cases and child-initiated in others. In either case, working for pay can be seen as an alternative form of extra-curricular activity that is voluntary. This contributes to the perception that working was at the parents’ discretion and that parents were *opting in* to the activity. The discretionary nature of working for pay appears to contribute to an overall sense of confidence in workplace safety and parental perception of control over occupational hazards.

### Being in control

Many parents stated that they felt in control of their young teen’s work situation and that they were taking personal responsibility for their son’s or daughter’s safety while on the job. It would seem that the perception of being in control mitigated any serious concerns about workplace safety and may explain why some parents expressed a complete lack of safety concerns. Related to the feeling of being in control, most parents stated that they would not have allowed their child to work if they thought the job was dangerous. This theme was well articulated by these two mothers:

*I don’t have any concerns. No. It’s a controlled environment, I feel like I’m in control of the situation because I am well aware of what’s happening and who it is happening with. So to me there are only positives. There are absolutely no negatives*. (Mother of 13 year old daughter, dance instructor).

*I don’t think I would let my son have a job if I believed there was too much risk involved.* (Mother of 14 year old son, ice cream shop).

The term *control* is used in part to reflect the language of the parents, but also to convey an overall sense of personal responsibility and ownership. Parents were not in a position to dictate the specific day-to-day tasks encountered by their young teens on the job, and therefore control in this case does not refer to micro-level oversight of the workplace. Rather, the theme of being in control related to a more strategic sense of having confidence in their 12 to 14 year olds’ wellbeing at work and the perception of responsibility for addressing any safety concerns associated with the job. No discernible thematic differences emerged according to job type with respect to an overarching sense of parental control; however, the level of formality inherent to their 12 to 14 year olds’ jobs was not explored in any significant depth.

### Custodial function of paid work

The presence of the aforementioned themes of familiarity, trust, discretionary authority, and perception of control appeared to overshadow any serious safety concerns while their young teens were at work. In contrast, parents expressed considerable concern about the potential risks to their young teens while unsupervised, such as during transit between home, work, and school, or while engaging in unrestricted leisure activities. In fact, the idea of 12 to 14 year olds having too much unrestricted free time emerged as a great concern for parents. Many parents noted that their young teen’s workplace was a known, supervised environment, thus implying safety. As stated by one mother of a 14 year old son, working "*helps to keep them out of trouble versus hanging out at the mall*". The notion that work could serve as a form of supervision in addition to school or other extracurricular activities was an underlying theme throughout the discussions. For some parents, it would appear that work took on a custodial function. Having their young teens occupied in a supervised environment seemed to provide additional sense of security and safety.

*When this job came about a year and a half ago, I hesitated at first. But then when I did all my homework and what not, it was a pretty good idea. She is never by herself, she’s got company, she can’t be bored. If anything happens I am a phone call away.* (Mother of 13 year old daughter, yard work & snow shoveling)

Not all parents espoused the custodial nature of part-time employment for their young teens, however. Some parents discussed actively monitoring their 12 to 14 year old on the job by providing cell phones for maintaining regular contact while babysitting or accompanying them on their newspaper route, such as described by this mother:

*My son never goes alone. It is always myself, his dad, his brother accompanies him. I would never let him go alone.* (Mother of 13 year old son, newspaper delivery)

Parental supervisory styles differed in response to the range of jobs held by these young teens. Parents seemed to adopt different monitoring behaviours depending on the level of formality inherent to their child’s job. In contrast to proxy-supervision behaviours such as those exemplified by the mother in the above quotation as well as the provision of cell phones, other parents entrusted all workplace supervision to their 12 to 14 year old’s employer.

*The owner of the store is always there in my case. There is supervision all the time. He is told what he has to do. Someone is there all the time.* (Father of 13 year old son, convenience store)

This suggests that parents are actively making assessments about the degree of supervision necessary in their 12 to 14 year old’s job, and then delegating responsibility accordingly. Regardless of whether this was a function of implicit trust or the sense of control over workplace safety, these themes served to reinforce the downplaying of occupational safety concerns in favour of the perceived benefits of part-time employment as a young teen.

## Discussion

This study focused on the perceptions and understandings of parents of working 12 to 14 year olds, an age group where many work for pay, but has received little attention in the workplace safety literature. Our findings suggest that parents of 12 to 14 year olds perceive many benefits to working at this age despite the potential risks associated with their young teens’ jobs. Parental feelings of responsibility for young teen safety and being in control of the work situation appear to contribute to a risk discounting process. Previous research with 12 to 14 year old workers has been primarily descriptive in nature. By contrast, this qualitative study provides explanatory insight into parental understandings and perceptions.

The resulting themes that emerged from this qualitative analysis give rise to two key questions: 1) why did parents in this study focus on the specific safety concerns of kidnapping and assault over and above other more probable work-related hazards?, and 2) why did parents of babysitters and newspaper deliverers in particular assume so much responsibility for their young teens’ occupational health and safety needs?

The way in which parents overemphasized certain risks, when probed, to the exclusion of others can be understood as a process of risk discounting. A useful theory in which to situate these findings can be found in Sandman’s [34] hazard and outrage risk perception model. This model proposes that the perception of risk is the product of an individual’s appraisal of a risky event and the strength of their response to that event, termed as *outrage*. Whereas hazard appraisal is a technical assessment of risk, outrage consists of the emotional and behavioural responses to risk and is thus socially constructed [35]. Sandman and colleagues have identified over 15 factors that moderate how risk is perceived [36, 34]. The qualitative themes emerging from this study suggest that parents discounted risk in at least six ways (i.e. familiarity, knowability and understanding, trust, control, voluntariness, and benefits), all of which are in parallel to the factors comprising Sandman’s [34] risk perception model.

Parents expressed a level of *familiarity* with their 12 to 14 year old’s workplace. The risk perception model states that risks stemming from activities that are familiar will be judged as more acceptable [36]. Fear of abduction notwithstanding, this may account for the lack of concern over risks of physical injury that may be incurred on paper routes, in food services, or working in a family business, for example. Further, it may explain why parents expressed comfort with their sons and daughters performing odd jobs such as cleaning, yard work, and babysitting because the tasks associated with these jobs would be familiar to parents in the context of daily household maintenance and caretaking. In contrast, parents who were not already familiar with the job their young teen currently held took the initiative to gain information about the job. In this sense, the jobs held by 12 to 14 year olds were perceived as *knowable and understandable*. Well-understood risks, or those that can be easily explained, will be perceived as less harmful than those that are poorly understood [36]. Additionally, the heightened vigilance associated with potential abduction or assault is explained by the fact that these occurrences are *unknowable.* Whereas safety hazards in the workplace can be identified and mitigated, child abduction or assault at the hands of strangers can never be truly foreseen. Many parents acknowledged that their fear of strangers permeated much of their parenting and was not limited to on-the-job concerns.

Implicit *trust* in employers and workplaces was a major theme throughout the focus groups. Activities associated with individuals or organizations deemed trustworthy will be perceived as less risky [36, 34]. As such, employers or settings deemed as trustworthy will be more readily acceptable to parents. Parents also repeatedly stated that they were firmly in *control* of their 12 to 14 year olds’ work situation. The risk perception model proposes that activities perceived to be under control of the individual will be judged as more acceptable [36], whereas activities under the control of others will appear to be of higher risk. In this case, parents downplayed potential hazards due to the perception of control. In a similar vein, activities undertaken *voluntarily* are much more likely to be judged as acceptable [36, 34]. As parents felt that they were allowing, and in many cases encouraging, their child to work for pay, any risk associated with the job would likely be downplayed. Finally, activities with clear *benefits* will generally be perceived to be lower in risk. Numerous studies have found an inverse relationship between risk and benefit [37]. Even if activities have identifiable risks, the presence of additional benefits to the individual will be viewed as more acceptable overall [36, 38]. It is clear that in this study, benefits were a mitigating factor in parental understanding and concerns over potential safety risks associated with their 12 to 14 year olds’ paid work. The cumulative nature of these factors likely serves to further strengthen parents’ risk discounting of common job hazards.

In contrast, the parents’ focus on low probability, severe risks (e.g., kidnapping and assault) are consistent with the cognitive bias called the availability heuristic [39]. The availability heuristic refers to the fact that judgments about the perceived likelihood of a future event are influenced by how frequently one hears about similar events. That is, the parents’ nearly exclusive concern about these low probability events may be partly explained by the fact that the media more often carries news stories about children being kidnapped than children sustaining work injuries performing odd jobs. Given that injury rates for 12 to 14 year old workers are comparable to those of older youth, it is important that parents have a more accurate understanding of the risks involved with their young teens’ jobs.

With respect to why certain parents assumed so much responsibility for the occupational health and safety of 12 to 14 year olds, we noted many examples of parents engaging in proxy supervision and personally implementing safety measures (e.g. providing cell phones, safety equipment, or safety training) throughout the focus group discussions. This was largely a function of the types of jobs held by 12 to 14 year olds in our sample, particularly those engaging in newspaper delivery and odd jobs, which are less likely to constitute a fixed work location or formal work agreement.

The consequences of these unofficial work arrangements in no fixed workplace is that the youth were perceived as being "self-employed." This status seemed to lead to an ambiguity about what duties and responsibilities for work safety those hiring the 12 to 14 year old were assuming. Key criteria for determining self-employment status of a worker in Canada and the U.S. includes degree of control over work tasks and ownership of equipment used on the job [26]. From the description provided by the parents in our sample, youth (and by extension the parent) had a substantial degree of control over when and/or where they worked. Most young teens working common odd jobs could arguably meet criteria for being self-employed or perceive themselves to be self-employed [26]. In many odd jobs this issue appears unclear, although we would hypothesize that a newspaper company would bear more responsibility than a homeowner in many of these cases. In any case, inthe absence of clearly defined work safety duties and responsibilities, it follows that parents would take on the additional responsibility of monitoring their 12 to 14 year old when working. Parents are essentially taking on a supervisory role in place of that which should be provided by a newspaper company or private homeowner.

Previous research with parents of older teenagers indicates that parents view paid work as an opportunity to teach work ethic, responsibility, and build character, and that these are skills not otherwise learned in school [40, 16]. Indeed, a surprising finding of the current study was the lack of discussion about the tradeoffs between time devoted to paid work and time spent on schoolwork, particularly given that so many 12 to 14 year olds were reported as working during the school year. Parents in this study appeared to support a developmental model of work/school balance, whereby paid work serves to further overall youth development and that the activities of work and schooling mutually reinforce each other [14].

Finally, the emergence of parental responsibility for supervision as a theme suggests that parents in this study understood safety to reflect a broader context within their children’s lives. Their young teen’s job may simply be viewed as one aspect of many in their life for which parents felt ultimately responsible. Again, this reflects the development stage of 12 to 14 year olds, differentiating them from older teenagers.

### Limitations

This focus group study was relatively small and included only those parents whose 12 to 14 year olds were currently working for pay. There may have been a selection effect in that parents with more favourable attitudes toward their young teenagers working may have been more likely to participate. Parents may also have sought to present socially desirable images of themselves as actively engaged in their children’s lives. The use of a market research company for recruitment may have resulted in a less representative sample than could have been achieved through random digit dialing. This strategy, however, did allow for a broader reach than the more traditional use of recruitment flyers posted in newspapers or public spaces. In addition, the focus group methodology was purposefully chosen to elicit a diverse range of opinions. As such, our results do not reflect the prevalence of different types of jobs held by 12 to 14 year olds in Ontario, nor the generalization of attitudes to all parents, even though common themes emerged across focus groups. Although efforts were taken to recruit a range of socio-economic and ethnic backgrounds, all focus group participants resided in an urban setting and the jobs held by their 12 to 14 year olds were non-agricultural in nature. Resulting themes therefore may not apply to parents of 12 to 14 year old farm workers, who would potentially emphasize a much different set of work-related hazards. Themes of responsibility and control, however, may remain relevant to these parents.

## Conclusions

These preliminary findings are not enough to provide specific guidance in terms of interventions or policies. Nevertheless, given the overemphasis by parents on rare safety concerns such as abduction, it would be useful to further investigate the other hazards more commonly encountered by 12 to 14 year olds in the workplace, and effectively communicate these risks to parents. A key implication of the apparent self-employed status of those working odd jobs such as news paper delivery is that policy makers and those responsible for child labour enforcement should clarify who is responsible for occupational health and safety in those work arrangements. The implications for future research include exploring among parents and the youth themselves more about the contextual factors that lead to risk appraisal and discounting of risk. It would also be important to explore the perspectives of employers with regards to understandings and perceptions of workplace safety for 12 to 14 year olds.
